# DDIT3 Expression in Liposarcoma Development

**DOI:** 10.1155/2014/954671

**Published:** 2014-03-25

**Authors:** Christina Kåbjörn Gustafsson, Katarina Engström, Pierre Åman

**Affiliations:** ^1^Sahlgrenska Cancer Center, Department of Pathology, Institute of Biomedicine, University of Gothenburg, P.O. Box 425, 40530 Gothenburg, Sweden; ^2^Department of Oncology, Institute of Medical Sciences, University of Gothenburg, Gothenburg, Sweden

## Abstract

Liposarcomas are mesenchymal tumors containing variable numbers of lipoblasts or adipocytes. The most common entities, well differentiated/dedifferentiated liposarcoma (WDLS/DDLS) and myxoid/round cell liposarcoma (MLS/RCLS), are both characterized by genetic rearrangements that affect the expression of the transcription factor DDIT3. DDIT3 induces liposarcoma morphology when ectopically expressed in a human fibrosarcoma. The role of DDIT3 in lipomatous tumors is, however, unclear. We have analyzed the expression of DDIT3 in 37 cases of liposarcoma (WDLS/DDLS *n* = 10, MLS/RCLS *n* = 16, and pleomorphic liposarcomas (PLS) *n* = 11) and 11 cases of common benign lipomas. Major cell subpopulations of WDLS/DDLS and MLS/RCLS tumors were found to express DDIT3 or the derived fusion protein, whereas PLS cases showed only a few positive cells. The lipomas contained large subpopulations expressing DDIT3. No correlation between numbers of DDIT3 expressing cells and numbers of lipoblasts/adipocytes was found. In vitro adipogenic treatment of two DDIT3 expressing cell lines induced lipid accumulation in small subpopulations only. Our results suggest a dual, promoting and limiting, role for DDIT3 in the formation of lipoblasts and liposarcoma morphology.

## 1. Introduction

Adipocytic tumors are the most frequent types of soft tissue tumors occurring in humans. They also represent the largest single group of mesenchymal tumors [1] and are characterized by the more or less prominent presence of lipoblasts or adipocytes. Some of the adipocytic neoplasms are characterized by recurrent tumor type specific genetic rearrangements. Most WDLS/DDLS cases contain amplified segments of chromosome 12q13–15 carried as ring chromosomes or large marker chromosomes [2–4]. The amplified regions contain many tumor associated genes and among them* DDIT3*. MLS/RCLS carry a rearranged* DDIT3* fused to* FUS* or* EWSR1* [5, 6].* DDIT3* is expressed and involved in the regulation of adipocyte development and we have previously shown that expression of* DDIT3* in a low differentiated fibrosarcoma cell line results in morphological conversion towards a liposarcoma phenotype [7]. These observations suggest that expression of DDIT3 protein could be a common phenotype determining factor for several types of lipomatous tumors. The aim of the present study was to test this hypothesis by investigating the expression of the DDIT3 protein in 3 different subtypes of liposarcoma and in common lipoma.

We further evaluated the role of DDIT3 expression by studying lipoblast formation in cultured liposarcoma cells treated with adipogenic factors. DDIT3 expression is tightly regulated at several levels including translation and protein degradation [8]. This makes expression analysis at protein level the most relevant approach.

## 2. Material and Methods

### 2.1. Immunohistochemistry

Paraffin embedded sections were obtained from our pathology department in conformity with Swedish legislation. The material consists of 11 lipomas, 11 PLS, 10 WDLS, and 16 MLS/RCLS.

Sections were prepared from routine paraffin embedded tumor tissue samples from 48 cases of lipomas and liposarcomas (Table 1). Immunohistochemistry (IHC) was performed as described previously [7] using the DDIT3 specific antibody Gadd153 R20 from Santa Cruz Biotechnology at a dilution of 1 : 200.

The histological specimens were examined and evaluated in a blinded fashion by two examiners. The proportion of stained tumor cells was counted at 200x magnification. Cells with nuclear and cytoplasmic expression were counted avoiding inflammatory cells, endothelial cells, and necrotic areas. Three different areas in each slide were counted and a mean value was calculated.

### 2.2. Fluorescence In Situ Hybridization Analysis

Interphase FISH analysis of formalin-fixed tumor tissue was performed on 1–4 *μ*m paraffin sections. Three break-apart probes, DDIT3, FUS, and EWSR1 (Vysis, Inc., Downers Grove, IL), were used according to protocols supplied by the manufacturer. Nuclei were counterstained with 10 *μ*L 4′,6′,-diamidino-2′-phenylindole dihydrochloride (DAPI). The sections were analyzed and reanalyzed by two independent reviewers. At least 100 nuclei per section were scored. The interpretation of intact, fusion, and split signals was based on guidelines recommended by the manufacturer and from other clinical laboratories using this method.

### 2.3. In Vitro Adipogenesis

The GOT3 cell line, established from a WDLS tumor [4], was used to study adipogenic differentiation. The cells were cultured in RPMI 1640 until 100% confluence, after which the medium was changed to adipogenesis induction medium (PT3004 containing human recombinant insulin, dexamethasone, indomethacin, and 3-isobutyl-l-methylxanthine (IBMX); Cambrex, East Rutherford, NJ) or maintenance medium (MM; RPMI 1640 containing 8% fetal calf serum). The cells were treated with adipogenesis induction medium for 3 days, followed by 1 to 3 days in MM. This was repeated three times. Control cultures were fed with only MM following the same schedule. After completed cycles, the cells were cultured for 7 more days in MM with replacement of the medium every 2 to 3 days. The cells were inspected using a microscope, and accumulation of fat was assessed by staining the cells with Oil Red O after fixation with 4% buffered formalin.

## 3. Results and Discussion

**Figure 1:**
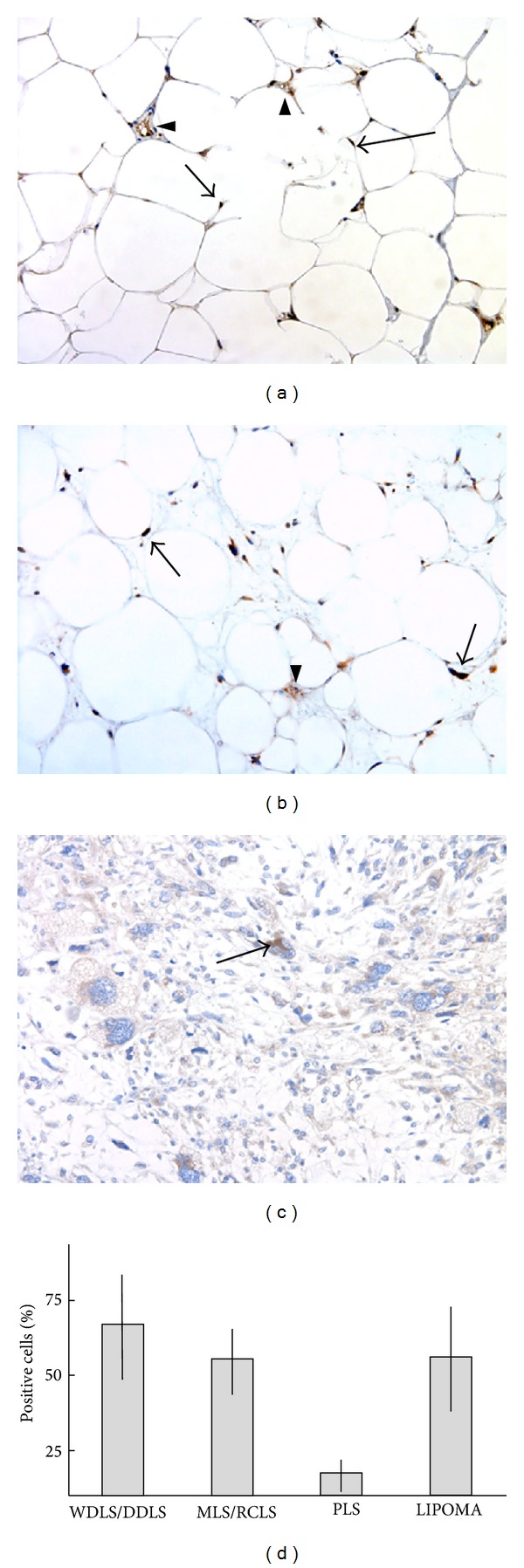
DDIT3 immunohistochemistry analysis of lipomatous tumors. (a) Lipoma, nuclear, and DDIT3 expression (arrow) and capillaries are indicated by arrowheads. (b) WDLS/DDLS, nuclear, and cytoplasmic DDIT3 expression (arrow). (c) PLS, nuclear, and cytoplasmic DDIT3 expression in lipoblasts (arrow). (d) Mean value and standard deviation of percent positively stained cells in 10 cases of WDLS/DDLS, 16 cases of MLS/RCLS, 11 cases of PLS, and 11 cases of lipoma.

Clinical data, histological features, and DDIT3 protein expression are detailed in Table 1 and representative examples of IHC staining are shown in Figure 1. DDIT3 expression was detected in all but 2 of the 48 investigated cases.

In WDLS/DDLS tumors 40–94% of the cells expressed DDIT3 (Table 1). The constitutive DDIT3 expression in this tumor type could be explained by recurrent gene amplicons that carry the DDIT3 in this tumor type [2, 4]. There was no obvious difference in numbers of DDIT3 expressing cells between WDLS and DDLS in this small series of tumors.

In MLS/RCLS, DDIT3 is expressed as part of the FUS-DDIT3 or EWSR1-DDIT3 fusion oncoproteins [5, 9]. Transcription of the fusion oncogenes is regulated by the ubiquitously active promoters of the* FUS* or* EWSR1* partner genes [6]. The MLS/RCLS tumors contained 24–73% FUS-DDIT3 or EWSR1-DDIT3 positive cells (Table 1). The fact that the fusion proteins could be detected only in a subpopulation of the tumor cells indicates that the levels of fusion proteins are regulated also at posttranscriptional levels. There was no clear difference in numbers of positive cells between typical myxoid cases and those with round cell components.

A cytoplasmic DDIT3 staining pattern was seen in most PLS cases but only in a low percentage of the cells. Three tumors (cases 29, 30, and 31) showed a nuclear staining pattern of DDIT3 in a minority of the cells, mostly with bizarre nuclei. There are no reported recurrent genetic aberrations causing DDIT3 expression in PLS. Instead, stress induced expression may explain the presence of DDIT3 protein in these tumors. Cytoplasmic or nuclear DDIT3 expression may be induced as a response to various stress conditions such as DNA damage, hypoxia, and lack of nutrients [10–12]. Such conditions are common in tumor tissues and may thus lead to DDIT3 expression.

The highly differentiated common lipoma expressed DDIT3 in 15–81% of the cells (Figure 1). The DDIT3 protein was found both in univacuolated fat cells and surrounding spindle cells.* DDIT3* is not amplified or rearranged in lipomas. Instead, most of the cases carry a rearranged* HMGA2* gene that may promote adipocytic differentiation [13]. The DDIT3 expression found in lipoma cells may therefore result from a terminal adipocyte differentiation program in these tumor cells [14]. Thus, there is currently no reason to believe that aberrant DDIT3 expression is involved in the development of this tumor type.

**Figure 2:**
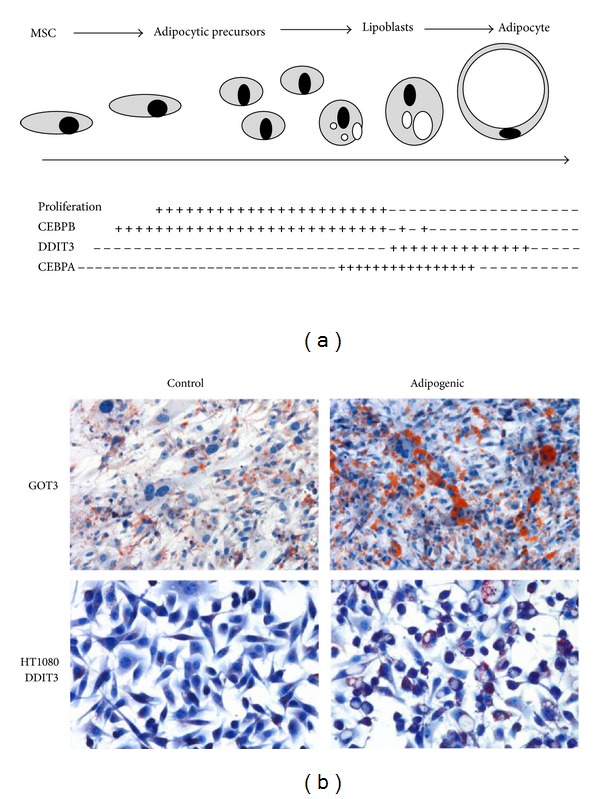
Adipocytic differentiation. (a) Schematic presentation of CEBPB, CEBPA, and DDIT3 expression in adipocyte differentiation. (b) Lipoblast formation and accumulation of lipids in GOT3 WDLS/DDLS derived cell line (top panels) and DDIT3 transfected HT1080 fibrosarcoma cell line (bottom panels) cultured in control or adipogenic medium. Oil Red O staining shows lipids in red.

DDIT3 is normally expressed late in adipocyte differentiation together with the related transcription factor CEBPA [14]. The timing of expression of DDIT3 and CEBP factors is crucial for normal differentiation (Figure 2). Premature or overexpression of DDIT3 in preadipocytes may block terminal differentiation [15–17]. In the context of liposarcoma development, aberrant DDIT3 or FUS-DDIT3 expression in primitive tumor cells may open an adipocytic differentiation pathway but block later stages of adipocyte development. We speculate that only a small minority of the tumor cells would make it through the block and differentiate to lipoblasts. This would also explain the lack of correlation between numbers of DDIT3 expressing cells and numbers of lipoblasts in the investigated tumors.

To test this hypothesis, the WDLS/DDLS derived cell line GOT3 was analyzed after adipogenic treatment. This cell line carries a large chromosome 12 derived amplicon including the DDIT3 gene and was found to express DDIT3 constitutively in almost all cells [4]. A limited accumulation of lipids in sporadic cells was seen under standard culture (Figure 2(b)). Transfer to adipogenic culture conditions resulted in lipid accumulation and lipoblast development but only in a minority of the cells. Similar results were obtained for a fibrosarcoma cell line stably transfected with EGFP tagged DDIT3 (Figure 2(b)). These results suggest that aberrant expression of DDIT3 can promote a liposarcoma phenotype in human primitive sarcoma cells [7].

*FUS-DDIT3* transfected mouse mesenchymal stem cells cause MLS/RCLS like tumors when injected in mice and* FUS-DDIT3* transfection transforms 3T3 mouse fibroblasts [18, 19]. This shows that* FUS-DDIT3* is a powerful oncogene. The FUS-DDIT3 protein maintains the capacity of DDIT3 to form heterodimers with CEBPA and CEBPB and has been shown to modify or block the activity of its dimer partners [17, 19]. Blocking of CEBPA could inhibit terminal differentiation [17]. Since transcription of the fusion gene is driven by the ubiquitously active FUS promoter, failed expression timing of this abnormal variant of DDIT3 may also contribute to the oncogenic activity. The FUS part of FUS-DDIT3 was found to be necessary for transformation of 3T3 fibroblasts [19] and forced expression of FUS-DDIT3 failed to induce cell cycle arrest as reported for DDIT3.

In contrast to FUS-DDIT3, forced expression of the DDIT3 protein in mesenchymal cells or in transgenic mice gave no evidence of transformation or tumorigenic activity [20]. The normal DDIT3 protein thus cannot be considered a driving oncoprotein. DDIT3 expression may, however, halt proliferation [19, 21]. In WLDLS and DDLS it is overexpressed together with several other proto-oncogenes such as MDM2 and CDK4 that together may cause tumor development. Aberrantly expressed, DDIT3 may in this context act as a promoting or/and tumor-type directing factor by blocking or interfering with the adipocyte differentiation program and the associated growth termination.

Originally described as a nuclear DNA-binding transcription factor, stress induced DDIT3 has later been reported to localize in the cytoplasmic compartment [21]. Cytoplasmic DDIT3 was shown to induce partially distinct effects compared to nuclear DDIT3. One proposed mechanism was sequestration of its CEBP family dimerization partners [21]. In the present study we observed cytoplasmic DDIT3 primarily in PLS cells containing bizarre nuclei, thus supporting a stress related mechanism behind the expression in these cells. Stress induced DDIT3 expression is more likely temporary but could anyway trigger some of the low differentiated PLS cells into an adipocytic differentiation path explaining the presence of lipoblasts in this tumor type. As in WLDLS and DDLS, expression of DDIT3 may also contribute to tumor development by halting the adipocytic program and associated growth termination.

In summary, DDIT3 is expressed in subpopulations of tumor cells in all 4 investigated lipomatous tumor types. There was no obvious difference between the number of DDIT3 expressing cells in the more aggressive DDLS and RCLS compared to WDLS and MLS. Furthermore, DDIT3 was expressed at comparable levels in benign lipomas. Only a minority of DDIT3 expressing sarcoma cells responded to adipogenic conditions in vitro indicating a complex role for DDIT3 as a phenotype directing factor in lipomatous tumors.

## Figures and Tables

**Table 1:** Cases and DDIT3 expression.

Case	Age	Site		DDIT3 %		FUS/DDIT3 rearrangement	Histological diagnosis
				Nucleus	Cytoplasm		
1	72	im	Axilla	79	0	ND	WLDS
2	69	im	Thigh	34	0	ND	WLDS
3	77	im	Hip	82	0	ND	WLDS
4	71	other	Retroperitoneal	0	36	ND	DDLS
5	42	other	Abdomen	70	0	ND	DDLS
6	80	other	Abdomen	94	0	ND	DDLS
7	73	other	Inguinal	40	0	ND	WDLS
8	77	other	Peritoneal	84	0	ND	WDLS
9	60	other	Inguinal	69	0	ND	WDLS
10	67	im	Thigh	81	0	ND	WDLS/DDLS
11	80	im	Thigh	59	0	Yes	MLS
12	34	im	Thigh	61	0	Yes	MLS
13	49	im	Hip	46	0	Yes	MLS
14	46	im/sc	Hip/thigh	71	0	Yes	MLS
15	39	im	Thigh	24	0	Yes	MLS
16	17	im	Thigh	39	0	Nev	MLS
17	45	im	Thigh	58	0	Yes	MLS
18	76	sc	Back	44	0	Yes	MLS/RCLS
19	42	im	Abdomen	70	0	Yes	MLS/RCLS
20	33	other	Abdomen	50	0	Yes	MLS/RCLS
21	45	other	Abdomen	41	0	Nev	MLS/RCLS
22	73	im	Leg	49	0	Nev	MLS/RCLS
23	38	im	Thigh	64	0	Yes	MLS/RCLS
24	36	im	Thigh	73	0	Yes	MLS/RCLS
25	37	im	Thigh	50	0	Yes	MLS/RCLS
26	46	im	Hip	0	57	Yes	RCLS
27	84	im	Arm	0	9	ND	PLS
28	57	im	Iliopsoas	0	0	ND	PLS
29	89	im	Leg	9	0	ND	PLS
30	88	im	Thigh	2	0	ND	PLS
31	70	im	Thigh	18	0	ND	PLS
32	72	im	Neck	0	7	ND	PLS
33	81	im	Axilla	0	6	ND	PLS
34	69	im	Thigh	0	1	ND	PLS
35	61	im	Thigh	0	9	ND	PLS
36	51	im	Hip	0	0	ND	PLS
37	54	im	Hip	0	20	ND	PLS
38	44	sc	Inguinal	53	0	ND	Lipoma
39	44	sc	Shoulder	54	0	ND	Lipoma
40	51	sc	Shoulder	68	0	ND	Lipoma
41	56	sc	Neck	32	0	ND	Lipoma
42	40	sc	Hip	40	0	ND	Lipoma
43	41	sc	Neck	15	0	ND	Lipoma
44	72	sc	Thigh	38	0	ND	Lipoma
45	38	sc	Arm	73	0	ND	Lipoma
46	66	sc	Arm	71	0	ND	Lipoma
47	66	sc	Thigh	81	0	ND	Lipoma
48	47	sc	Back	75	0	ND	Lipoma

Clinical, diagnostic, and DDIT expression data on the investigated cases. DDIT3 expression is shown as percent positive cells.

Abbreviations: im: 0 intramuscular; sc: subcutaneous; ND: not analyzed; nev: not possible to evaluate.
